# Evaluating Quality Management and Diagnostics Microbiology Performance Within an International External Quality Assessment (EQA) Program Serving National One Health Sector Reference Laboratories Across Asia: Experience Amid the Coronavirus Disease 2019 (COVID-19) Pandemic

**DOI:** 10.1093/cid/ciad569

**Published:** 2023-12-20

**Authors:** Freshwork Ayalew Abegaz, Patrícia Teixeira dos Santos, Ondari D Mogeni, Tobin Guarnacci, Nimesh Poudyal, Jinhui Hong, Soo Young Kwon, Rikke Braae, Rangsiya Prathan, Taradon Luang Tongkum, Watcharaporn Kamjumpho, Rungtip Chuanchuen, Florian Marks, Rene S Hendriksen, Marianne Holm

**Affiliations:** International Vaccine Institute, Seoul, Republic of Korea; The Technical University of Denmark, National Food Institute, WHO Collaborating Center for Antimicrobial Resistance in Foodborne Pathogens and Genomics and European Union Reference Laboratory for Antimicrobial Resistance, FAO Reference Laboratory for Antimicrobial Resistance, Kgs. Lyngby, Denmark; International Vaccine Institute, Seoul, Republic of Korea; International Vaccine Institute, Seoul, Republic of Korea; International Vaccine Institute, Seoul, Republic of Korea; International Vaccine Institute, Seoul, Republic of Korea; International Vaccine Institute, Seoul, Republic of Korea; The Technical University of Denmark, National Food Institute, WHO Collaborating Center for Antimicrobial Resistance in Foodborne Pathogens and Genomics and European Union Reference Laboratory for Antimicrobial Resistance, FAO Reference Laboratory for Antimicrobial Resistance, Kgs. Lyngby, Denmark; Research Unit for Microbial Food Safety and Antimicrobial Resistance, Faculty of Veterinary Science, Chulalongkorn University, Bangkok, Thailand; Research Unit for Microbial Food Safety and Antimicrobial Resistance, Faculty of Veterinary Science, Chulalongkorn University, Bangkok, Thailand; National Institute of Health, Bangkok, Thailand; Research Unit for Microbial Food Safety and Antimicrobial Resistance, Faculty of Veterinary Science, Chulalongkorn University, Bangkok, Thailand; International Vaccine Institute, Seoul, Republic of Korea; Cambridge Institute of Therapeutic Immunology and Infectious Disease, University of Cambridge School of Clinical Medicine, Cambridge, United Kingdom; Heidelberg Institute of Global Health, University of Heidelberg, Heidelberg, Germany; Madagascar Institute for Vaccine Research, University of Antananarivo, Antananarivo, Madagascar; The Technical University of Denmark, National Food Institute, WHO Collaborating Center for Antimicrobial Resistance in Foodborne Pathogens and Genomics and European Union Reference Laboratory for Antimicrobial Resistance, FAO Reference Laboratory for Antimicrobial Resistance, Kgs. Lyngby, Denmark; International Vaccine Institute, Seoul, Republic of Korea

**Keywords:** external quality assessment, remote assessment, antimicrobial resistance, one health, quality management system

## Abstract

**Background:**

Strengthening external quality assessment (EQA) services across the One Health sector supports implementation of effective antimicrobial resistance (AMR) control strategies. Here we describe and compare 2 different approaches for conducting virtual laboratory follow-up assessments within an EQA program to evaluate quality management system (QMS) and procedures for pathogen identification and antimicrobial susceptibility testing (AST).

**Methods:**

During the coronavirus disease 2019 (COVID-19) pandemic in 2021 and 2022, 2 laboratory assessment approaches were introduced: virtual-based and survey-based methodologies. The evaluation of 2 underperforming Animal Health laboratories through a virtual-based approach occurred between May and August 2021. This evaluation encompassed the utilization of 3 online meetings and document reviews, performed subsequent to the execution of EQA procedures. Within a distinct group of laboratories, the survey-based assessment was implemented from December 2021 to February 2022, also following EQA procedures. This phase encompassed the dissemination of an online survey to 31 participating laboratories, alongside a sole online consultation meeting involving 4 specific underperforming laboratories.

**Results:**

The virtual-based assessment post-EQA aimed to identify gaps and areas for improvement in the laboratory's practices for pathogen identification and AST. This approach was, however, time-intensive, and, hence, only 2 laboratories were assessed. In addition, limited interactions in virtual platforms compromised the assessment quality. The survey-based post-EQA assessment enabled evaluation of 31 laboratories. Despite limitations for in-depth analysis of each procedure, gaps in QMS across multiple laboratories were identified and tailored laboratory-specific recommendations were provided.

**Conclusions:**

Reliable internet and plans for efficient time management, post-EQA virtual laboratory follow-up assessments are an effective alternative when conducting onsite evaluation is infeasible as observed during the COVID-19 pandemic, although the successful implementation of remediation plans will likely require in person assessments. We advocate application of hybrid approaches (both onsite and virtual) for targeted capacity building of AMR procedures with the ability to implement and oversee the process.

## Teaser

External quality assessment (EQA) is crucial for quality assurance in antimicrobial resistance (AMR) surveillance and effective control interventions. Optimized capacity-building approaches are needed post-EQA for different scenarios, including pandemics with travel restrictions.

The “Global Action Plan on AMR,” jointly developed by the World Health Organization (WHO), the World Organization for Animal Health (WOAH), and the Food and Agriculture Organization (FAO), emphasizes the threat of antimicrobial resistance (AMR) as a global human, animal, and environmental health security issue [1]. This requires coordinated multisectoral collaboration and implementation for the Sustainable Development Goals (SDGs) to be met [2, 3]. Generally, AMR affects all countries but disproportionately impacts resource-limited settings.

Ensuring effective AMR response demands robust surveillance across the One Health (OH) sector [4]. Good quality bacteriology laboratory and well-functioning AMR laboratory networks are critical to generating accurate and consistent surveillance data supporting policy development and effective AMR control strategies [5]. Health systems in many developing countries face, however, challenges, including inadequate microbiology laboratory facilities and quality-assured AMR diagnostics capacity due to infrastructure and human resource constraints [6]. To alleviate these challenges, external quality assessment (EQA) programs evaluate and provide laboratories with information about overall competency levels and training needs [7]. As such, periodic laboratory quality assessments are crucial to ensure quality and reliable AMR surveillance laboratory data [8].

To strengthen the provision of EQA services across the OH sector among National Microbiology Reference Laboratories/Centers of Excellence in South and Southeast Asia, the UK AID Fleming Fund Regional Grant “EQAsia” was launched in January 2020. The Project aimed to increase the quality of laboratory-based surveillance of WHO GLASS pathogens and FAO-priority pathogens through regional EQA providers [9]. In the inception phase of EQAsia, the coverage, availability, and uptake of EQA programs across the OH sector in South and Southeast Asia were mapped [10]. The EQA coverage levels and quality oversight structures were heterogeneous across countries, especially OH sectors, with a wide range of capacity levels and readiness to participate in EQA schemes [10]. These findings supported the implementation phase design and introduction of a standardized, comprehensive EQA program for AMR to all National Reference Laboratories (NRLs) across the OH sector in the Asian region, which was rolled out in 3 EQA rounds in 2021 [9, 11].

As part of capacity-building activities, virtual workshops were conducted, encompassing quality management system (QMS) standards, basic and advanced microbiological techniques for pathogen identification and AMR diagnostics, as well as specific elements of EQA provision and participation. As part of EQA, the EQAsia Consortium virtually conducted a follow-up assessment of selected underperforming labs to identify gaps and areas for improvement in the laboratory practices and to inform other specific capacity-building needs. Due to border closure during the coronavirus disease 2019 (COVID-19) pandemic, follow-up activities were conducted virtually.

In this study, we compare 2 different approaches to conducting virtual laboratory follow-up assessments within an EQA program, evaluating both the QMSs, pathogen identification, and antimicrobial susceptibility testing (AST) performance.

## METHODS

Following the inception phase of the EQAsia program in August 2020, baseline symposiums were conducted. Based on their existing capacity levels identified during the inception phase, NRLs from 10 Fleming Fund countries (Pakistan, Nepal, Bhutan, Bangladesh, Vietnam, Laos, Sri Lanka, Indonesia, Timor-Leste, and Papua New Guinea) and non-Fleming Fund countries (Brunei, Philippines, and Maldives) were invited to participate in the EQAs and relevant training workshop modules.

### Training Workshops for the National Reference Laboratories

Two virtual workshops were conducted over 1–2 weeks, each focusing on the effective and safe EQA participation and requirements for EQA provision, basic and advanced QMSs, basic and advanced pathogen identification, and AST. Participants received an email with a ZOOM link at least a week before the session.

The first workshop was conducted from January to February 2021 in the preceding EQAs. It included modules introducing laboratory QMSs, pathogen identification, and basic AST procedures, including disk diffusion, broth microdilution, and gradient strip tests. Similarly, a module focused on the general requirements for setting up an EQA scheme, including knowledge of the ISO17043:2010, was delivered to NRLs at a more advanced stage of EQA participation/provision. The second workshop was conducted in May 2021. In addition to the modules provided in workshop 1, it also included sessions on more advanced elements within QMSs and microbiology diagnostics (eg, techniques in polymerase chain reaction and whole-genome sequencing).

### External Quality Assessment of National Reference Laboratories

**Figure 1.**
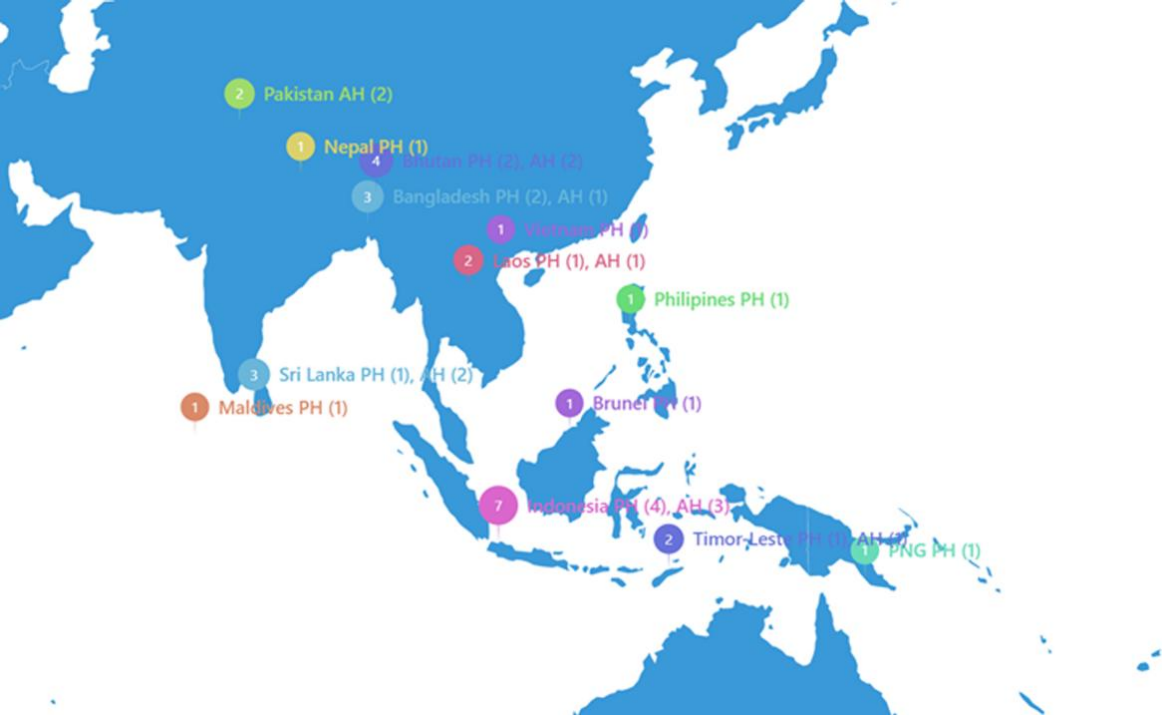
Public and Animal Health laboratories participated in the first and second rounds of EQA. Numbers in parenthesis indicate the number of labs that participated in EQA. Abbreviations: AH, Animal Health; EQA, external quality assessment; PH, Public Health; PNG, Papua New Guinea.

The procedures and results of the EQAs preceding the virtual-based and survey-based assessments, including a Matrix EQA (selective isolation of presumptive ESBL-, AmpC-, and carbapenemase-producing *Escherichia coli* from complex matrices, such as food) is published elsewhere [11, 12]. In the first iteration, 8 strains of each organism (*Salmonella* and *E. coli)* were selected based on phenotypic AMR profile to include a heterogeneous panel, allowing for strain variation from almost pan-resistant to fully susceptible isolates. After preparation and external validation, strains were lyophilized to be propagated and shipped to 23 laboratories registered in the EQAsia EQA program preceding the virtual-based assessment. Similarly, in the second iteration, panels containing eight strains of *Klebsiella pneumoniae*, *Shigella* spp., *Acinetobacter* spp., and *Staphylococcus aureus* were distributed to 24 laboratories in the EQA program preceding survey-based assessment (Figure 1). In total, 9 laboratories registered for and participated in the EQAsia Matrix EQA.

### Selection of Underperforming Laboratories

The participants' performance in the EQA was evaluated based on the European Committee on Antimicrobial Susceptibility Testing (EUCAST) and the Clinical and Laboratory Standards Institute (CLSI) guidelines [13]. Results in agreement with the expected interpretation were categorized as “1'” (correct), whereas results deviating from the predicted performance were classified as “0'” (incorrect). The selection of underperforming laboratories was based on 3 criteria: the ability to identify bacterial pathogens, the AST of quality control strains, and the AST of test strains.

Among 23 laboratories that participated in the EQA preceding the virtual-based assessment, six Animal Health laboratories were identified as underperforming. Considering the pilot nature of the virtual-based assessment and anticipated limitations of remote support to underperforming laboratories, the 2 least underperforming Animal Health NRLs were chosen for the virtual-based assessment.

Initially, we planned to conduct an onsite follow-up assessment by a team of external assessors from the EQAsia consortium to local, underperforming laboratories. Due to the COVID-19 pandemic and travel restrictions, a protocol was, however, developed outlining the process for virtual laboratory assessments.

Two different remote follow-up assessments were conducted after each EQA. The virtual-based assessment following EQA was conducted through a series of online meetings between May and August 2021 for 2 underperforming Animal Health laboratories. The survey-based assessment following EQA was conducted between December 2021 and February 2022. It consisted of an online survey of 31 participating laboratories and single online consultation meetings with 4 underperforming (3 Public and 1 Animal health) laboratories.

### Procedures for the Virtual-Based Assessment of the Underperforming Laboratories

For the follow-up assessment of the 2 underperforming laboratories, an assessment team was formed composed of relevant members and experts from each EQAsia Consortium partner. The assessment team developed assessment questionnaires according to the ISO17025:2017 and CLSI guideline QMS01-A4; Quality Management System: A Model for Laboratory Services; Approved Guideline—Fourth Edition.

#### Pre-assessment Activities

The opening meetings were held with the first laboratory on 27 May 2021 and the second on 9 June 2021. These sessions outlined the assessment's scope, date, and communication preferences. Laboratories were asked to establish a secure document-sharing system for essential materials such as quality manuals, standard operating procedures (SOPs), internal audits, quality control, personnel files, equipment calibration, and maintenance records. Subsequent to these meetings, standardized assessment questionnaires were sent along with virtual assessment notifications to NRLs. The questionnaire addressed issues related to facility and security, organization and management, personnel training, and evaluation aspects. Additionally, it encompassed quality assurance, document control, equipment maintenance, EQA, sample handling, inventory management, and Information technology (Supplementary File 1). The laboratories were given 10 business days to respond to the questions in the questionnaire.

Furthermore, labs were required to prepare bacterial cultures before the virtual assessment, facilitating real-time AST analysis on assessment day.

#### The Virtual Laboratory Assessment

In the allotted 4-hour virtual laboratory assessment, we attempted to conduct a remote laboratory walk-through covering the 3 phases of laboratory processes—pre-analytical, analytical, and post-analytical processes, assessing AST procedures and conducting in-depth video interviews with subject matter experts. The first lab was evaluated on 26 June 2021, and the second from 15 to 16 July 2021. Subsequently, a remote review of shared documentation including quality manuals, SOPs, equipment records, and the questionnaire was conducted, adhering to predefined assessment procedures.

#### Post-assessment Activities

**Figure 2.**
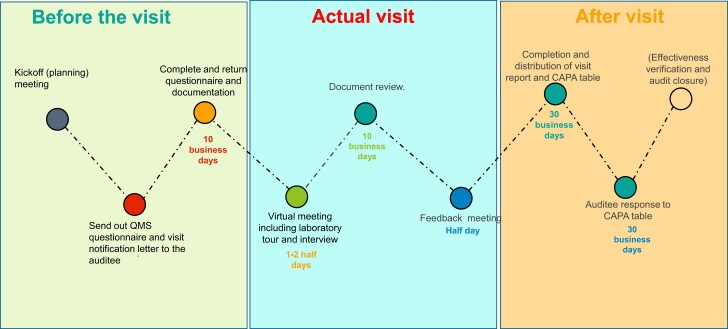
Procedures of the virtual-based assessment. Abbreviations: CAPA†, corrective and preventive action; QMS*, quality management system.

After assessment, feedback meetings were held on 9 July 2021 and 27 August 2021 for the respective labs. Prior to these meetings, summaries of observations from virtual tours, document reviews, and interviews with subject matter experts (microbiologists and quality managers) were shared (Figure 2). In these sessions, the assessment team presented findings, proposed recommendations, and received the labs' responses. Each assessment's outcome was detailed in a narrative report and a corrective and preventive action (CAPA) table, later distributed to participants. Labs were also given the CAPA table for their responses, including root-cause analysis, corrective and preventive actions, timelines, and designated personnel for addressing CAPAs.

### Procedures for the Survey-Based Assessment Post EQA

#### Survey Development and Dissemination

**Figure 3.**
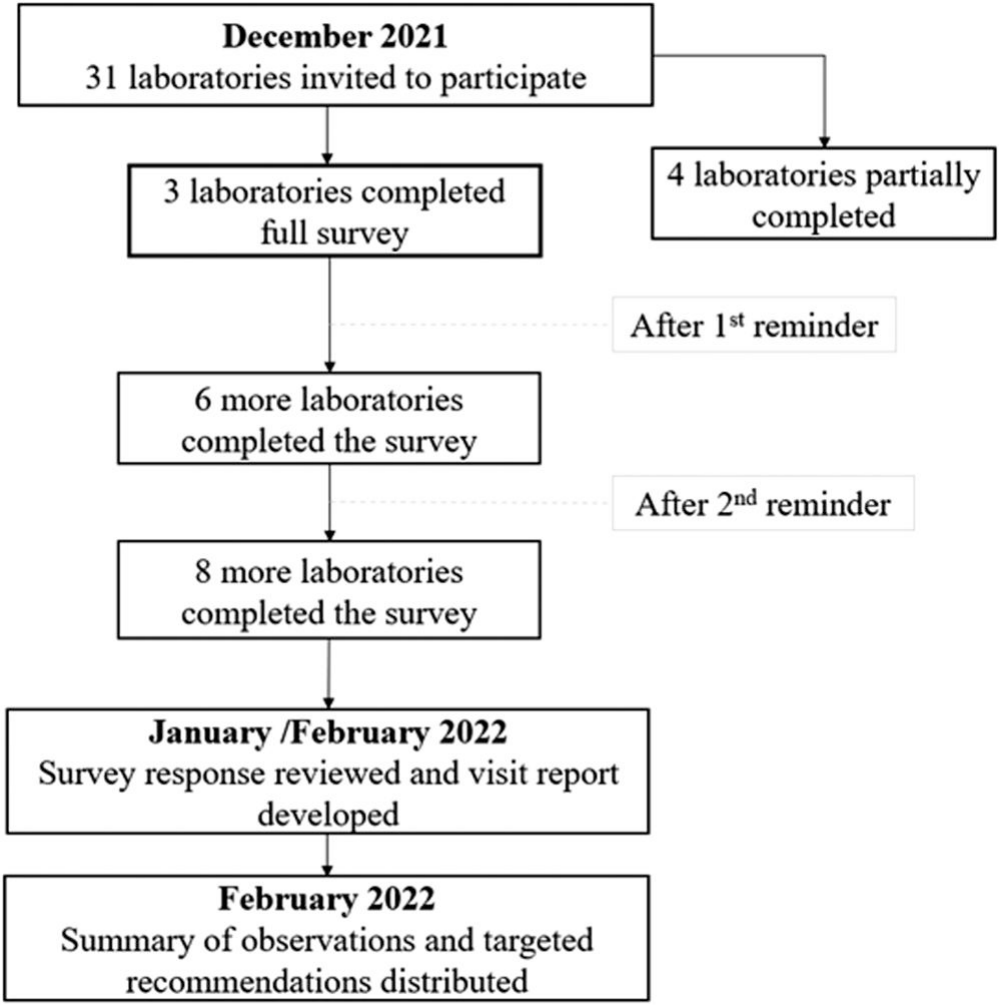
Procedure of the survey-based assessment.

For the survey-based assessment, an online QMS survey questionnaire (Supplementary file 2) was developed and disseminated to all 31 laboratories registered to participate in EQA (of which 24 submitted EQA results). This survey consisted of the same questions in the virtual-based assessment. The elements of the survey questionnaire were assembled based on ISO 15189:2012, CLSI, and ISO 17027:2017. They consisted of 111 questions related to (1) organization and management, (2) quality assurance/quality management, (3) document control/SOPs, (4) personnel training and competency assessment, (5) internal quality control/EQA, (6) equipment calibration and maintenance, (7) specimen collection and handling, (8) reagent and consumables management, and (9) methods used for collection, processing, and storage of data. In December 2021, an email invitation containing a survey link and purpose explanation was sent. Figure 3 depicts participating labs' survey responses .

For a more in-depth evaluation of pathogen identification and AST performance, a flexible approach was employed compared to the virtual-based assessment. Consultation meetings were held with the 3 Public and 1 Animal health underperforming laboratories that participated in the EQA preceding survey-based assessment. Each laboratory was contacted before the consultation meeting to arrange a time and date for the 1-hour online session and a supporting document review inclusive of all observations, which were shared with the participating laboratories to assist in preparation for discussion. During the online meeting, The EQAsia team presented observations, addressed discrepancies in EQA trials, discussed consumables and techniques, and explored potential root causes for inaccurate results. Following the laboratories' responses, the EQAsia team and the respective laboratory identified documentation and practical examples that could help support the laboratory's improvement and participation in future EQAs.

### Data Analysis

Survey responses were collected using the Survey Method. They were then exported from the Survey Method into Excel workbook (*.xlsx) format.

## RESULTS

### Virtual-Based Assessment

Remote interviews, document reviews, and virtual laboratory tours revealed a lack of established policies and procedures for laboratory performance. Participating laboratories demonstrated inconsistent documentation of Quality Management System (QMS) activities, notably lacking records for internal audits, corrective/preventive actions, and annual management reviews. A review of pre-recorded videos highlighted deviations from international guidelines (ISO, CLSI) in handling lab materials and preparing antimicrobial stock solutions. Moreover, AST results obtained for certain antimicrobials indicated that the lab was using expired antimicrobial disks. A virtual laboratory visit revealed that the pH of the media was not checked, no purity control for media, and non-compliance with McFarland standard preparation. To address these gaps, supportive references and recommendations were incorporated into the CAPA tables fostering continual laboratory improvement.

### Survey-Based Assessment

Out of the initially registered 31 laboratories for EQA2, 24 laboratories participated and 55% (17/31) responded to the online survey (Figure 2), with 6 from Animal Health and 11 from Public Health sectors. Analysis of survey data revealed that among the 17 surveyed laboratories, 24% (4/17) had not implemented a Quality Management System (QMS). Additionally, 47% (8/17) lacked established policies, and Procedures for tasks such as internal audits, non-conforming work management, and quality control programs. Furthermore, 60% (9/17) lacked SOPs for root-cause analysis and corrective/preventive actions. Annual management reviews and internal audits were not conducted in 41% (7/17) of the laboratories. Notably, 47% (8/17) displayed a gap in reagent and consumables management, lacking quality control checks for new reagents and consumables prior to use. Documentation for reagent evaluation was also deficient in these cases.

Among the surveyed laboratories, poor documentation processes for personnel training were noted in 41% (7/17), while the absence of SOPs and schedules for equipment function checks, preventive maintenance, and calibration were noted in 41% (7/17) laboratories. All labs participated in EQA for pathogen identification and AST in at least one EQA program, but 47% (8/17) lacked procedures to support required proficiency testing. Additionally, 47% (8/17) revealed an absence of documentation as evidence of corrective actions taken for EQA deviations (Table 1). From the four underperforming laboratories invited for the consultation meeting for pathogen identification and AST, a common issue was the lack of quality control procedures, failure to test the recommended quality control strains, and incorrect inoculum preparation.

**Table 1.** Gaps Identified During the Virtual-Based and Survey-Based Assessments

Elements of the Questionnaire	Identified Gaps	
	Virtual-based assessment	Survey-based assessment
Quality assurance/Quality management	No process was observed to be available for performing root cause analysis The audit tool (ie, checklist prepared based on international standards) was not available No record of non-conforming work, corrective action, and management review was provided for review The Laboratory Quality Manual was not available	In 4 of 17 labs, QMS was not implemented 7 of 17 labs, an Annual management review, and an internal audit were not conducted No audit tool is available in 7/17 labs 6 of 17 labs, a Quality manual was not available No record of corrective/preventive action was available in the 8/17 labs In 10 of 17 labs, environmental conditions were not monitored
Document control/SOPs	Before the effective date, no process was observed for personnel training to new or updated controlled documents No document was observed to be associated with the name and signature of the person authoring the document The format of the SOP did not include the scope, responsibility, and glossary/definition Lack of compliance with Good Documentation Practice, that is, use of “line down” and may black space be observed in the equipment monitoring log No document was observed to be associated with the name and signature of the author, reviewer, and approver of the document No SOPs related to the laboratory procedures being performed were observed to be available for laboratory staff	8 of 17 labs, SOPs for conducting an internal audit, identifying, and managing non-conforming work, and an internal quality control program were unavailable
Personnel orientation, training, and Assessment	No process was observed for the orientation and training of new employees No SOP training log was observed in the personnel record No training materials, curriculum, or certificates were observed in the personnel record supporting the training No competency record was provided for review	7 of 17 labs, SOPs for employee training were not available 7 of 17 labs, training log was not maintained 5 of 17 labs, training materials, curriculum, and certificates were unavailable 5 of 17 labs, competency assessment was not conducted
Sample shipping, receipt/processing	No specimen rejection log was provided for review for the specimen that does not fulfill the criteria for acceptance	5 of 17 labs, specimen rejection logbook was absent
Proficiency testing	No approved procedure to support PT testing (ie, peer analysis) was available for review	In 8 of 17 labs, no approved procedure to support PT testing (ie, peer analysis) was available
Equipment calibration and maintenance	No maintenance, operation, and calibration procedures were available for all equipment Micropipettes are subject to annual calibration, which is contrary to industry guidance and best practice which recommends quarterly (ie, every 3 months) calibration cycles Calibration documentation was not available for all equipment No equipment handbook was available near each piece of equipment The equipment maintenance record was not maintained	7 of 17 labs, SOPs for equipment containing sufficient detail regarding methods, materials, and schedules to be used in routine inspection, cleaning, maintenance, testing, and calibration of equipment, were absent 3 of 17 labs, Equipment used for measurement and testing were not calibrated 2 of 17 labs, inventory of equipment was not maintained
Reagent storage and control	No Inventory records for reagents and consumables Access to the storage room is not controlled	4 of 17 labs, SOP for the reception, storage, acceptance testing, and inventory management of reagents and consumables were absent 8 of 17 labs, QC check was not performed for the new shipment, and the unique lot number of reagent and consumables
Electronic system and IT	No documentation supporting internally developed laboratory management system software had been subject to regulatory requirements for software system validation and management No SOP existed for all computer system uses, operations, and maintenance No training manual for computer system application No training was provided for all staff on the use of computer system	11 of 17 labs implement a laboratory information system (LIS) to manage and report results 2 of 11 labs, laboratory information management system software had not been subject to regulatory requirements for software system validation and management 6 of 11 labs, SOPs for all uses, operations, and maintenance of the computer system and training manual did not exist 4 of 11 labs, no training for all staff on the use of all computer systems was provided and documented

Abbreviations: IT, information technology; PT, proficiency testing; QC, quality control; QMS, quality management system; SOP, standard operating procedure.

### Challenges and Opportunities During Follow-up Visit Procedures

A virtual-based assessment was conducted between May 2021 and August 2021 with two underperforming Animal Health laboratories. This follow-up aimed to comprehensively evaluate their practices, identify gaps and enhance pathogen identification and AST quality. The process involved remote interviews, document reviews, and virtual tours. The follow-up process was labor-intensive and time-consuming and the ability to directly interact with only two laboratories due to time and resource constraints. During the remote virtual laboratory meeting, a virtual video tour of the laboratory and supporting facilities was requested to evaluate the facility, equipment, and laboratory operations. A detailed walk-through in all the interested areas was, however, not possible due to low video quality and poor internet connection. Another challenge was language barriers in one of the laboratories. Even though the meeting was conducted with the help of an Interpreter, there was communication difficulty, especially with sharing the requested documents. Furthermore, none of the laboratories managed to complete the CAPA response following the completion of the visit.

Ultimately, in addition to only reaching two laboratories, the insufficient virtual interactions and challenges in communication meant these follow-up exercises were unlikely to have achieved the intended high-quality assessment to realize quality improvement and support laboratories in improving their performance.

The survey-based assessment involved distributing an online QMS survey to all participating labs. A follow-up evaluation included more labs (n = 17), given the more flexible approach. Although this mode of assessment was more generic across all participating laboratories and did not allow for an in-depth evaluation of individual laboratories, the survey identified key QMS gaps across laboratories. It should be noted that establishing reliable survey responses requires a detailed laboratory-specific “site visit”.

An online consultation meeting with four underperforming laboratories found significant gaps in the accuracy of pathogen identification and AST. The laboratories did, however, not provide adequate responses to the observations that were forwarded by the EQAsia team. Furthermore, the participants response requires onsite verification and not all subject matter experts attended the meeting.

Table 2 summarizes the challenges and opportunities of the two approaches in identifying pathogen identification and AST practice gaps and improvements.

**Table 2.** Challenges and Opportunities of the Virtual-Based and Survey-Based Assessments

	Virtual- Based Assessment	Survey-Based Assessment
Approach	Remote/virtual laboratory assessment	Online QMS Survey Questionnaire prepared and administered
Duration	From May to August 2021	From December 2021 to February 2022
Number of laboratories assessed	Two laboratories	Seventeen laboratories, of which four were selected for online consultation meetings
Opportunities	The laboratories were receptive to our recommendations and eager to learn and improve	The online survey enabled us to include and assess a larger number of laboratories Helped identify key gaps and develop a tailored recommendation based on observations The online consultation meetings helped us identify significant gaps in pathogen identification and challenges for performing AST properly
Challenges	Poor internet connection Low video quality Language barrier Time-consuming CAPA response was not completed	Did not allow for tailored, in-depth evaluation of individual laboratory's quality management systems Establishing the reliability of the survey response requires validation (eg, through individual site visits) Recommendation more generic Not all subject matter experts attended the consultation meetings Lack of adequate response for the observations shared through the supporting documents Reliability of the participant's response to the assessment team's questions requires onsite verification

Abbreviations: AST, antimicrobial susceptibility testing; CAPA, corrective and preventive action; QMS, quality management system.

## DISCUSSION

The EQAsia project implemented structured EQA assessments, including performance ratings, after providing a virtual capacity-building and training program to participating laboratories. In this article, we described the procedures for evaluation and outcomes of 2 modes of remote follow-up assessments conducted after 2 rounds of EQA to NRLs across the OH sector in South and Southeast Asian countries. Based on the data collected from remote interviews, review of laboratory documentation, and virtual laboratory tours, the most significant gap identified was the lack of supporting documentation as evidence of the activities being conducted. This was also reported in laboratory evaluations where no quality control records or summaries of corrective actions were maintained [14]. Another identified challenge hindering laboratory improvement was a lack of policies and procedures to support efficient, effective, high-quality laboratory operations and services. According to the survey responses from the second EQA participants, the most difficult problem was the lack of written internal quality control and corrective/preventive action supporting the remediation of non-conformities.

Routine quality control checks ensure the accuracy and reliability of test results. The lack of a quality control program in the laboratories identified in this paper is critical as it suggests a deficiency in laboratory leadership to implement regular internal quality control and corrective action programs. This shortcoming was similarly reported in laboratories where poor identification and AST performance were observed due to a lack of internal quality control and regular corrective actions [14, 15]. In addition, the lack of proper and attributable documentation of procedures potentially leads to inconsistency and inaccuracy in laboratory results [16].

Sustained engagement in EQA programs results in improved testing quality and performance of participating laboratories in pathogen identification and AST techniques [17]. The improvement in laboratory performance relies on in-depth analysis and identification of sources of errors that result in poor performance and are promptly addressed by continuous follow-up of the laboratories [18]. A key reason for poor performance in our study setting is related to inadequate training, poor adherence to standard protocol (eg, following CLSI guidelines for media antibiotic preparation, the inclusion of control strains), and the lack of human resources for rechecking and interpreting AST results. This is possibly related to improper strain handling and storage (contaminated, stored at room temperature or higher than −80C, and failure to follow ATCC storage methods), which may have resulted in reduced viability or contaminated strains. However, expired antimicrobial disks or improper media preparation (no pH measurement, too thick or too thin agar on plates) could also account for these findings. Another common cause of deviation was incorrect inoculum preparation, incorrect McFarland standard preparation, or the use of an expired commercially obtained McFarland standard. Misinterpretation and use of laboratory interpretive criteria were common, resulting in incorrect AST results despite the correct MIC value or inhibition zone diameter.

The different follow-up assessment approaches implemented in the EQAsia project represent options that can be applied when conditions for onsite laboratory visits are not feasible (such as during the COVID-19 pandemic). Although virtual laboratory assessment saves travel costs and provides an alternative option for evaluating the quality of testing and performance of laboratories, remote assessment requires additional time to identify gaps and areas for improvement in laboratory practices. In addition, it is more difficult to establish the reliability of findings without direct, onsite assessment. Furthermore, poor connectivity during the virtual laboratory tours greatly diminishes the quality and amount of information collected for evaluation and the establishment of efficient communication between laboratories, which is crucial. Even though attempts to enhance the assessment with a comprehensive document review and to complement the remote virtual tour with additional interviews with key stakeholders, also presented challenges related to time management and resources to the assessment exercise.

The survey-based approach of distributing survey questionnaires enabled reach to more laboratories. However, although it helped to identify critical gaps in laboratory systems and generate recommendations for all laboratories, verifying the validity of the laboratory responses was difficult. And the recommendations also ended up being more generic.

WHO’s Global Foodborne Infections Network (GFN) supports nations in enhancing integrated lab-based surveillance for foodborne and enteric infections through a One Health approach [19]. Although the WHO conducted the GFN program in the region from 1999 to 2015, capacities rapidly disappear if not maintained. These gaps hindered reliable data reporting to WHO and FAO, with the chance of taking the wrong actions or, worse, not taking action. Steps taken include revising assessment times to assess needs and proficiency and encouraging the global health actors to address the gaps, ensuring actionable surveillance data.

## CONCLUSION

In conclusion, the lessons learned from the EQAsia project revealed that follow-up capacity-building activities could be provided to laboratories in resource-limited settings using virtual assessments when conducting onsite evaluation is infeasible as observed during the COVID-19 pandemic, although the successful implementation of remediation plans will likely require in-person assessments. A virtual system, however, requires the establishment of a reliable internet connection and structured methodologies to ensure the adequate use of time and resources. Therefore, implementing a hybrid of approaches (both onsite and virtual) to capacity-building is recommended in consideration of time requirements and the careful consideration of efficient communication.

## Supplementary Data

Supplementary materials are available at *Clinical Infectious Diseases* online. Consisting of data provided by the authors to benefit the reader, the posted materials are not copyedited and are the sole responsibility of the authors, so questions or comments should be addressed to the corresponding author.

## Supplementary Material

ciad569_Supplementary_Data

## Notes

***Disclaimer.*** The views expressed here are those of the authors and not necessarily those of the UK Department of Health and Social Care or its management agent, Mott MacDonald; and, International Vaccine Institute.

The authors’ gratitude goes to external partners, the United States Food and Drug Administration (FDA), for validating the candidate EQA strains.

We also extend our appreciation to reference laboratory participants who engaged in virtual-based and survey-based assessments.

***Supplement sponsorship.*** This article appears as part of the supplement “Capturing Data on Antimicrobial Resistance Patterns and Trends in Use in Regions of Asia (CAPTURA),” funded by the Department of Health and Social Care's Fleming Fund using UK aid.

***Financial support*.** This work was supported by the Fleming Fund regional grants (grant number: FF64-474). The Fleming Fund is a UK aid investment to support surveillance of antimicrobial resistance in low- and middle-income countries in Africa and Asia. The regional grants program is managed by Mott MacDonald Ltd, the Fleming Fund Grants Management Agent.
