# Supplementary material for: Evaluating Quality Management and Diagnostics Microbiology Performance Within an International External Quality Assessment (EQA) Program Serving National One Health Sector Reference Laboratories Across Asia: Experience Amid the Coronavirus Disease 2019 (COVID-19) Pandemic
Source: Clin Infect Dis. 2023 Dec 20;77(Suppl 7):S588–96. doi: 10.1093/cid/ciad569 (PMC10732555; doi:10.1093/cid/ciad569)
Supplement: ciad569_Supplementary_Data [file ciad569_supplementary_data.zip › Online survey assessment questionnaire.pdf]

SurveyMethods
ASK. ANALYZE. IMPROVE.

Analyze Survey Results - Individual Results

Survey: QMS assessment questionnaire

Respondent Information		Custom Fields
Respondent Type:	Accessed Survey On Web	
Email:	No Email Available	
Response Status:	Completed PARTIAL Survey	
Survey URL:	https://app.surveymethods.com /EndUser.aspx?B490FCE3B6F6E4E6BF	
Began Survey On:	04/05/2022	
Time Began:	02:52:24 AM	
Time Spent:	00:00:39 (HH:MM:SS)	
Points:	No Points Questions used in this survey.	

1. **By participating in this Laboratory Quality Management System assessment questionnaire developed for the EQASIA project(as part of the Fleming Fund Grants Programme managed by Mott MacDonald), you are agreeing for your responses to be stored and utilized primarily for EQASIA project activities. The responses may also be shared with other Fleming Fund stakeholders and partners for purposes relating to other Fleming Fund activities.**

For details on Mott MacDonald’s privacy policy, please see the link to the website: www.mottmac.com/privacy-policy.

This questionnaire encompasses a total of 111 questions and covers the following section:

A. Facility and Security
B. Organization and Management
C. Personnel, Orientation, Training, and Assessment
D. Quality Assurance/Quality Management
E. Doc control/Standard Operating Procedures (SOP)
F. Sample shipping, receipt/Processing
G. Specimen Collection, Handling, and Reporting
H. Proficiency Testing
I. Glassware/Quality of Water/Reagents
J. Equipment calibration and Maintenance
K. Quality Control
L. Inventory Management
M. Electronic system and IT

Please note that answering all the survey questions takes approximately 15-30 minutes of your time. In case you are unable to complete the survey in one go, there is an option to save and complete later using the “save and resume” button at the end of each page.

I agree and understand the above statement.

2. **Name of the person responding**

3. **Email of the person responding**

4. **Name of laboratory (or affiliated institution/hospital)**

5. **Laboratory Information**

6. **1. Is access to the laboratory restricted?**

7. **2. How is entry monitored?**

8. **3. Is a security staff available 24 hours a day?**

9. 4. What are the back up service if a power failure occurs?

10. 5. Does the laboratory have a separate room for specimen collection, media preparation, sample preparation for microbiological analysis and reference testing.

11. 6. Is a Biosafety cabinet available and properly used for handling specimens?

12. 7. If yes, is the biologic safety cabinet certified at least annually to ensure that filters are functioning properly and that airflow rates meet specifications?

13. 8. Are environmental conditions checked and recorded daily for the following equipment?

	Yes	No	Do not know
Room temperature:			
Humidity:			
Freezers:			
Refrigerator:			
Incubators:			
Water Bath:			

14. 9. Is an appropriate fire extinguisher available, properly placed, in working condition, and routinely inspected? If yes, please write frequency of inspection

15. 10. What type of testing does the laboratory do?(Scope of operations)

16. 11. Is there a current laboratory quality manual, composed of the quality management system's policies?

17. 12. If yes, has the content of the quality manual been communicated to, understood and implemented by all staff?

18. 13. Do you have an organizational chart that shows:
- The organizational and management structure of the laboratory
- The laboratories place in any parent organization
- The relation between the laboratory, management, technical operations, support services, and the quality management system

19. 14. Who in the laboratory has overall responsibility for the technical operations and the provision of resources needed to ensure the required quality of laboratory operation?

20. 15. How many personnel are employed in your lab?

21. 16. Is there a training SOP defining processes for new employee training and orientation, internal and external training?

22. 17. Does each employee receive training on new and updated procedures and policies , quality management system, assigned work process, procedure and task?

23. 18. Is this documented in writing to include dates of training, type of training and the signature of the employee and trainer?

24. 19. Are competency assessment performed according to defined criteria (for new hires, existing staff) and documented?

25. 20. If yes, please describe how your laboratory assess and documents employee competency on the tasks they routinely perform

26. 21. Are training records readily retrievable in a manner that enables one to determine what training an employee has received, which employee has been trained on a particular procedure or have attended a particular program?

27. 22. Are records of personnel file which include job description, job orientation, previous work experience (CV), competency assessment, review of staff performance, education, and personnel qualification etc. maintained?

28. 23. Does the laboratory have a documented quality management (QM) system?

29. 24. Does the quality management system (QM) program follow a documented operational plan (This plan may be based upon some reference resource such as CLSI GP-22, ISO 15189 or ISO 17025 series, for improving organizational performance)?

30. 25. Does the laboratory summarize and review its records of errors and incident reports at defined intervals to identify trends and initiate corrective and preventive action (CAPA) as appropriate?

31. 26. Does the laboratory management perform a review of the quality management system at a management review meeting at least annually (There must be documentation that the laboratory director or designee (s) reviews the program regularly)?

32. 27.Are findings and actions from the management review communicated to the relevant staff?

33. 28. Is there a quality officer/manager with delegated responsibility to oversee compliance with the quality management system?

34. 29. Do you have policies and procedures for conducting internal audits to verify that the activities carried out in the laboratory comply with the requirement of the quality management system?

35. 30. Is internal auditing performed to confirm that all activities in the SOPs of the laboratory are being followed as written?

36. 31. Does your laboratory have an audit tool(checklist prepared based up on international standards) for conducting internal audit?

37. 32. Are the personnel conducting the internal audits trained with proven competency in auditing managerial and/or technical requirements?

38. 33. Does the quality SOP specify the scope and frequency of audits and how such audits are to be documented?

39. 34. Are internal audit findings presented to the laboratory management and relevant staff for review?

40. 35. Does the laboratory have a procedure for identification and control of non conforming work?

41. 36. Is documented root cause analysis performed for non- conforming work before corrective actions are implemented?

42. 37. Does a formal Corrective and Preventive Action (CAPA) procedure exist to ensure observation identified during routine auditing are associated with Root cause analysis, CAPA, effectiveness verification, and CAPA closure, management and tracking?

43. 38. Are implemented corrective actions monitored and reviewed for their effectiveness before closure/clearance?

44. 39. Is there a documented process for creating, reviewing , updating SOPs?

45. 40. Are policies and/or SOPs for laboratory functions, available, current ,and approved by authorized personnel?

46. 41. Are all quality management procedures, forms and records maintained under document control ?

47. 42. Is there a documented process for ensuring that all staff are trained on pertinent SOPs and receive refresher training when SOPs are updated?

48. 43. How are SOPs handled when they are no longer needed?

49. 44. Are all SOPs established by the authority of the management? Is a historical life of SOPs, and all revisions, including the dates of such revisions maintained (i.e., master list available)?

50. 45. Does each laboratory area have immediately available laboratory manuals and SOPs relative to the laboratory procedures being performed(i.e., SOP for media preparation, pathogen identification and AST)?

51. 46. Does the laboratory routinely perform a documented review of all quality and technical records including but not limited to?

	Yes	No	Do not know
Environmental monitoring log sheets:			
Specimen rejection records:			
Equipment calibration and maintenance records:			
IQC records across all test areas:			
Outcomes of PTs and other forms of Inter-laboratory comparisons:			
Quality indicators:			

52. 47. Is there an archiving system that allows for easy and timely retrieval of archived records and results?

53. 48. Is there a dedicated samples or isolates receiving area in your laboratory(i.e., shipping/ receiving, and accessioning area)?

54. 49. Is there a documented procedure for receiving, accessioning and storage of isolates/specimens that were shipped from hospital or collection site?

55. 50. Where testing does not occur immediately upon arrival in the laboratory, are specimens stored appropriately prior to testing(i.e., at correct temperature for sample type, in non- frost-free freezers in controlled areas of the laboratory)?

56. 51. Are personnel responsible for specimen handling and processing routinely trained to follow these procedures?

57. 52. Is the laboratory open to receive samples and for testing beyond regular working hours?

58. 53. Are incoming samples adequately identified?

59. 54.Upon receipt what document are checked against to samples to ensure that all samples are present?

60. 55. Are all samples or isolates accompanied by a test requisition up on delivery to the laboratory?

61. 56. How are primary samples or isolates stored after initial analysis?

62. 57. How does the sample receipt group handle samples that are broken in transit, quantity not sufficient (QNS) or shipped in at the wrong temperature (i.e., do you have specimen acceptance criteria)?

63. 58. How are samples handled that are mislabeled or unlabeled?

64. 59. Will sample testing be held up if pertinent information is missing on the test requisition (i.e., demography, test requests, etc.)?

65. 60. How is missing information related to the isolate/ samples obtained?

66. 61. Do you have a specimen rejection logbook to register samples that do not fulfill specimen acceptance criteria?

67. 62. Does your laboratory collect specimen on site?

68. 63. Is there a documented procedure manual or other source for the complete collection and handling instructions of all laboratory specimens?

69. 64. Does the specimen collection manual include instructions for all the following elements as applicable)? preparation of the patient

- Type of collection container and amount of specimen to be collected
- Types and amounts of preservatives or anticoagulants
- Need for special handling between the time of collection and time received by the laboratory (e.g., refrigeration, immediate delivery)
- Proper specimen labeling
- Need for appropriate clinical data, when indicated

70. 65. Are specimens uniquely identified to minimize sample mix-ups, mislabeling, etc.?

71. 66. Are copies or files, reported results retained by the laboratory in a manner that permits prompt retrieval of the information?

72. 67. Are laboratory records and materials retained for an appropriate time defined by the laboratory(i.e.,2 years or more)?

73. 68. Are the laboratory's procedures for proficiency testing written and sufficient for the extent and complexity of testing done in the laboratory?

74. 69. Is there appropriate documentation of problems and their solutions identified by the proficiency testing system?(e.g., Corrective and Preventive action)?

75. 70. Is there a policy that prohibits interlaboratory communication about proficiency testing samples until after the deadline for submission of data to the proficiency testing provider?

76. 71. Is there a policy that prohibit referral of proficiency testing specimens to to another laboratory and interlaboratory

communication? test not performed if reference laboratory used?

77. 72. How often in year does your laboratory participate in EQA for AST?

78. 73.Is the performance of the laboratory in the PT program reviewed and discussed with relevant staff?

79. 74. If deviating results are obtained in EQA what follow-up done/measure is taken to address the nonconformance?

80. 75. Is there a written evidence that all problems identified by proficiency testing and alternative performance assessment have been recognized and corrected?

81. 76. Is glassware routinely inspected for cracks, chips, etc.?

82. 77. What is the process for ensuring that glassware is clean and free of contaminant and detergent residues?

83. 78. Do you prepare reagent grade water in your laboratory?

84. 79. If yes, is the reagent grade water tested for purity?

85. 80. Is there a documented statement of polices and procedures that defines the standard for, and frequency of testing water quality?

86. 81. Does the laboratory have appropriate equipment available for performing pathogen identification and AST?

87. 82. Is back up equipment available if a piece of equipment fails?

88. 83. Are all equipment and methods validated/verified on-site upon installation and before use and is documented evidence available?

89. 84. Do the SOPs for equipment describe remedial action to be taken in the event of failure or malfunction?

90. 85. Are the manufacturer's operator manuals for all equipment readily available to testing staff and, available in the language understood by staff?

91. 86. Is equipment used for measurement, testing (i.e., micropipette, thermometer, centrifuge, balance, water bath, incubator etc.) adequately tested, calibrated, and/or maintained?

92. 87. Do the SOPs for equipment contain sufficient detail regarding methods, materials, and schedules to be used in routine inspection, cleaning, maintenance, testing, calibration of equipment, and designate the person responsible for the performance of each operation?

93. 88. Does the laboratory have a calibration procedure for calibration dependent equipment (pipettes, centrifuges, balances, pH meter and thermometers)?

94. 89.Is routine user preventive maintenance performed on all equipment and recorded according to manufacturer's minimum requirements?

95. 90. Is the current equipment inventory data available for all equipment in the laboratory including the following information?
- Name of equipment
- Manufacturers or authorized supplier contact details
- Condition received (new, used, reconditioned)
- Serial number
- Date of receiving
- Date of entry into service after validation/ verification

96. 91. Are equipment operated by trained, competent and authorized personnel?

97. 92. Is non-functioning equipment appropriately labelled and removed from the laboratory or path of workflow following the equipment management policies and procedures?

98. 93. Is there a written QC program that defines procedures for determining analytic performance, establishments of limits, frequency of controls, and corrective action for QC data?

99. 94. Is internal quality control performed, documented, and verified for pathogen identification and AST tests/procedures before releasing patient results?

100. 95. Does the laboratory use specific bacterial control strains to assure the quality of media for bacterial cultures?

101. 96. Is each new lot of susceptibility disks checked for activity before use?

102. 97. How frequently does the laboratory test Quality Control strains? Please write frequency of quality control testing (i.e., every day, every week or every month)

103. 98. Are Quality Control records are available?

104. 99. At what interval does the Quality Control records reviewed (i.e., monthly, quarterly, annually) ?

105. 100. What remedial actions are taken if unacceptable errors are discovered during review?

106. 101. Is there documentation of corrective action taken when quality control results exceed the acceptable range or reviews identify non conformities in a timely manner?

107. 102. Does the laboratory have a procedure for the reception, storage, acceptance testing and inventory management of reagents and consumables?

108. 103. Does the lab maintain records for each reagent and consumable that contributes to the performance of examinations? (e.g., identity of the reagent, batch code or lot number, date of receiving, expiry date, date of entering service etc..)?

109. 104. Are inventory records complete and accurate, with minimum and maximum stock levels denoted and monitored?

110. 105. Is adequate cold storage available?

111. 106. Are storage areas set up and monitored appropriately (temperature, humidity)?

112. 107. Are storage areas access-controlled?

113. 108. Are all reagents/test kits in use (and in stock) currently within the manufacturer-assigned expiration or within stability?

114. 109. Is each new reagent preparation, new lot number, new shipment of reagents or consumables verified before use and documented? If yes, please explain the procedure below

115. 110. Is First Expiration-First-Out (FEFO) practiced (Do you store products that will expire first in front of products with a later expiry)?

116. 111. Does your laboratory implement laboratory information system (LIS) or laboratory information management system (LIMS) to manage and report results?

117. 112. Is a computer system validated?

118. 113. Is an individual identified as responsible for the computer system?

119. 114. Is there security in place to limit access to all computer applications and prevent intrusions?

120. 116. Do SOPs exist for all uses, operation, and maintenance of the computer system?

121. 117. Is there a training manual for all computer applications?

122. 118. Is there documented training for all staff on the use of all computer systems?
